# Dense Deposit Disease and C3 Glomerulopathy

**DOI:** 10.1016/j.semnephrol.2013.08.002

**Published:** 2013-11

**Authors:** Thomas D. Barbour, Matthew C. Pickering, H. Terence Cook

**Affiliations:** * Kidney Research UK, Centre for Complement and Inflammation Research, Imperial College London, London, United Kingdom; † Department of Rheumatology, Centre for Complement and Inflammation Research, Imperial College London, London, United Kingdom; ‡ Department of Pathology, Centre for Complement and Inflammation Research, Imperial College London, London, United Kingdom

**Keywords:** C3 glomerulopathy, dense deposit, complement

## Abstract

C3 glomerulopathy refers to those renal lesions characterized histologically by predominant C3 accumulation within the glomerulus, and pathogenetically by aberrant regulation of the alternative pathway of complement. Dense deposit disease is distinguished from other forms of C3 glomerulopathy by its characteristic appearance on electron microscopy. The extent to which dense deposit disease also differs from other forms of C3 glomerulopathy in terms of clinical features, natural history, and outcomes of treatment including renal transplantation is less clear. We discuss the pathophysiology of C3 glomerulopathy, with evidence for alternative pathway dysregulation obtained from affected individuals and complement factor H (Cfh)-deficient animal models. Recent linkage studies in familial C3 glomerulopathy have shown genomic rearrangements in the *Cfh-related* genes, for which the novel pathophysiologic concept of Cfh deregulation has been proposed.

In 1962 the renal pathologist, Berger, and electron microscopist, Galle, published the first description of dense deposit disease (DDD), based on needle biopsies performed in three patients at the Necker Hospital in Paris.^1^ They later extended their observations to a total of 14 patients.^2^ DDD is a rare lesion, so named because of the appearance on electron microscopy (EM) of extremely dark, ribbon-like, electron-dense material in the central layer (lamina densa) of the glomerular basement membrane (GBM). Deposits also may occur in the mesangium, Bowman’s capsule, and tubular basement membrane. DDD has variable morphology on light microscopy (LM), with a mesangioproliferative^3^ or membranoproliferative^4^, ^5^ appearance being most common. Other patterns including endocapillary proliferative and crescentic are described in both native disease and post-transplant recurrence.^6^ DDD is therefore a more accurate name for this lesion than membranoproliferative glomerulonephritis (MPGN) type 2.^7^ Immunofluorescence (IF) or immunohistochemistry (IHC) typically shows intense glomerular C3 staining with little or no immunoglobulin (Ig).^8^ However, this appearance is not specific for DDD, with one early French series of 49 patients with MPGN comprising 15 patients with isolated or predominant glomerular C3 staining, of whom only 5 patients had intramembranous dense deposits characteristic of DDD.^9^ In a 2007 French series of 19 patients, Servais et al^10^ introduced the term *C3 glomerulonephritis* (C3GN) to describe isolated glomerular C3 without intramembranous deposits. Accordingly, the term *C3 glomerulopathy* was proposed^11^ for all glomerular lesions, including DDD, that are characterized by predominant C3 accumulation within the glomerulus using IF/IHC. The presence of predominant or isolated C3 accumulation suggests activation of the alternative pathway (AP) of complement, and many cases of C3 glomerulopathy show genetic or acquired AP dysregulation.

Recognizable entities within C3 glomerulopathy include those with characteristic morphologic appearances (DDD and electron-dense intramembranous GBM transformation) and those with clear etiology (complement factor H-related 5 [CFHR5] nephropathy and the presence of an abnormal CFHR5 protein). Because postinfectious GN typically is characterized by a reversible decrease in serum C3 and the presence of glomerular C3 without Ig, differentiation from C3 glomerulopathy sometimes may be possible only with knowledge of the clinical course. In a number of cases in which there has been persistent renal disease after diagnosis of postinfectious GN, AP abnormalities have been detected and subsequent biopsy specimens were consistent with C3 glomerulopathy.^12^, ^13^, ^14^, ^15^ This includes a report of a young girl with acute post-streptococcal GN and low serum C3 levels in whom recurrent macroscopic hematuria prompted biopsy examinations 9 and 20 months after the initial presentation.^12^ The biopsy specimens showed MPGN, with IHC showing capillary wall and mesangial C3 without Ig or C1q, and EM showing intramembranous and mesangial deposits. A heterozygous sequence variant in the *CFHR5* gene, in which duplication of a single nucleotide resulted in a premature stop codon, later was identified in the girl and her unaffected mother. Notwithstanding some common pathogenetic associations, atypical hemolytic uremic syndrome (aHUS) is not considered a C3 glomerulopathy because endothelial injury usually is seen without significant C3 deposition or electron-dense deposits.^16^

## Histologic Features

**Figure 1:**
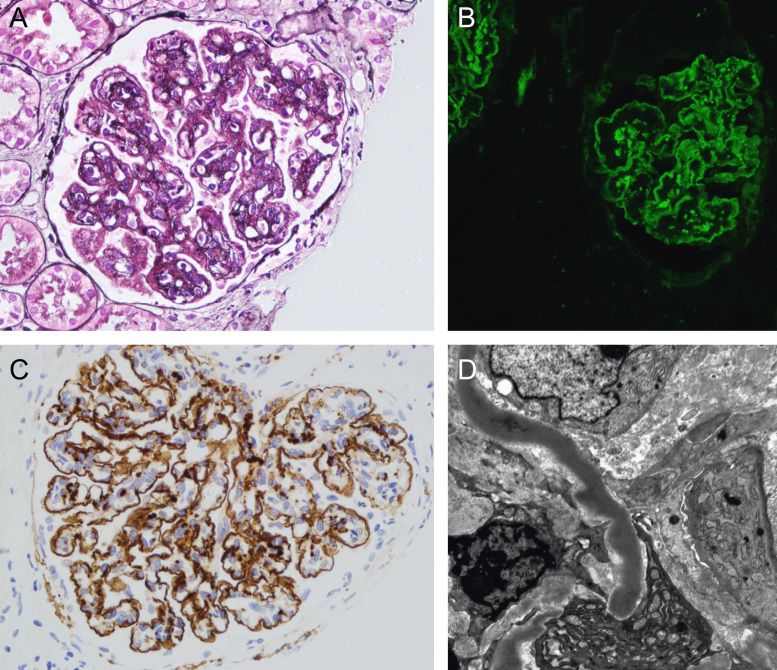
Renal histology in individuals with DDD. (A) Light microscopy with silver stain showing a membranoproliferative pattern with double contours of the GBM. (B) Immunofluorescence and (C) immunohistochemistry with immunoperoxidase showing strong capillary wall staining of C3 and some granular mesangial C3. (D) Characteristic sausage-like, intramembranous, osmiophilic deposits on electron microscopy. Reprinted with permission from Dr. C. Nast.

Electron-dense deposits are seen within the glomerulus in all forms of C3 glomerulopathy. DDD is defined by the intramembranous location of these deposits, their intensely osmiophilic appearance forming ribbons, and the associated transformation of the GBM (Fig. 1). Confirmation of DDD requires EM, although the diagnosis can be suspected with a high degree of confidence if the typical LM and IF/IHC features are present. The distinction between DDD and C3GN is sometimes difficult even using EM,^17^, ^18^, ^19^ with debates about the extent of intramembranous deposits required for EM diagnosis of DDD.^20^ Furthermore, because the ribbons of DDD typically are discontinuous and may be more prominent in some capillary loops than others, they may be missed as a result of biopsy sampling error, leading to a diagnosis of C3GN. Such discrepancies may be important in the context of prognostic or therapeutic studies comparing outcomes in DDD and C3GN.

**Figure 2:**
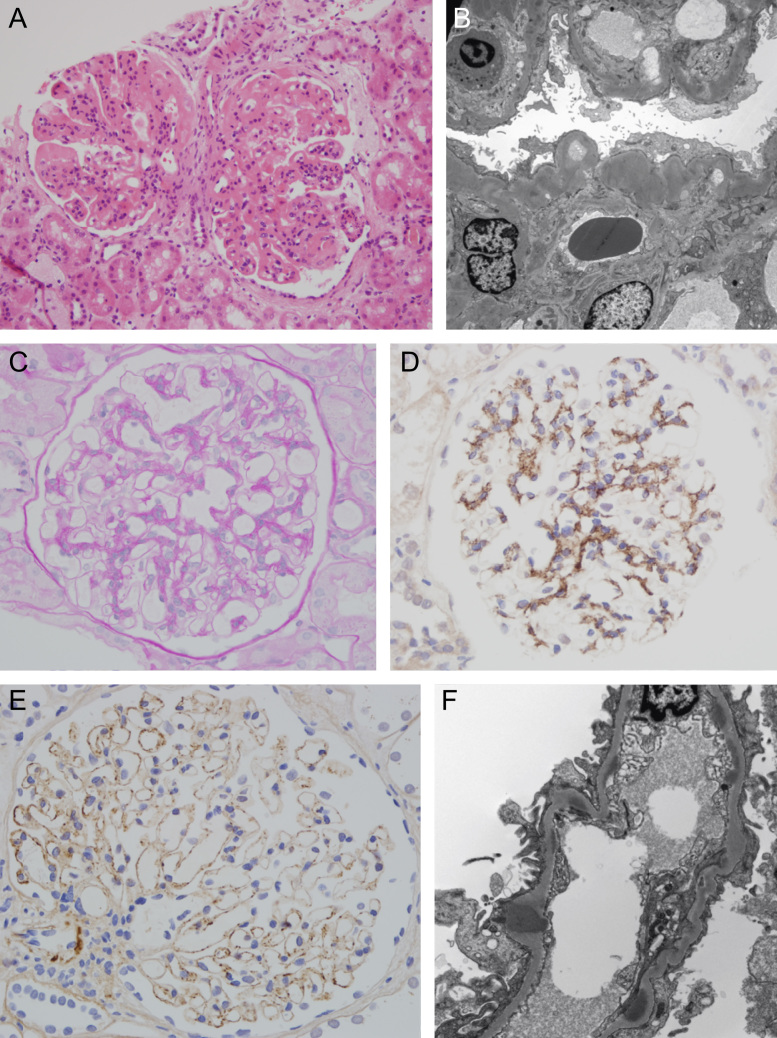
Renal histology in individuals with C3GN. (A) Light microscopy showing a membranoproliferative pattern with segmental endocapillary neutrophil infiltration. (B) Electron microscopy showing moderately dense capillary wall and mesangial deposits. (C) Light microscopy showing mesangial expansion and (D) immunoperoxidase showing mesangial C3 in the same individual. (E) Immunoperoxidase showing granular capillary wall C3, in this case without conspicuous mesangial deposits, and (F) electron microscopy showing predominantly subendothelial deposits (and a transmembranous deposit) in an individual with CFHR5 nephropathy.

The ultrastructural characteristics of C3GN are less clearly defined, other than by the absence of the hallmarks of DDD (Fig. 2). In the original French C3GN series by Servais et al,^10^ two main histologic groups were described. The first group had typical features of MPGN type 1, with mesangial proliferation, double contours, and subendothelial, mesangial, and (less commonly) subepithelial deposits. The second group lacked mesangial proliferation, a membranoproliferative pattern, or subendothelial deposits. EM was performed in only two patients (confirming the absence of dense intramembranous deposits).^10^ This series recently was updated to provide data on 29 DDD patients, 56 C3GN patients, and 49 MPGN type 1 patients (both adults and children).^21^ Occasionally, the EM appearance of C3GN closely resembles that previously described as a subtype of MPGN type 3 by Strife et al.^22^ In CFHR5 nephropathy, histologic appearances (Fig. 2E and F) include mesangial and/or capillary wall C3 deposits on IF; a normal or mesangioproliferative pattern on LM; and mesangial, subendothelial, and occasional subepithelial deposits of only moderate density on EM. Subepithelial deposits may in fact be present in all types of C3 glomerulopathy, sometimes with so-called humps identical to those characteristically seen in postinfectious GN. In one DDD series, paramesangial subepithelial deposits correlated with low serum C3 owing to C3 nephritic factor (C3NeF) activity^23^ and were composed of C3c.^24^ Laser microdissection and mass spectrometry of glomerular tissue has shown a similar proteomic profile for DDD^25^ and C3GN^17^ that includes alternative and terminal pathway complement components.

## Clinical Features

DDD has an estimated prevalence of 2 to 3 per million population^26^ and traditionally is viewed as a diagnosis of childhood and young adulthood.^27^ Several series have shown a significantly younger mean age at diagnosis among DDD patients compared with MPGN types 1/3^28^, ^29^, ^30^ or C3GN.^21^ However, some DDD cohorts have comprised numerous older patients,^31^ including one recent US series of 18 adults and 14 children in which 39% of adults were older than age 60 at diagnosis.^4^ Although this study and several others^30^, ^32^, ^33^, ^34^ found that DDD was almost twice as common in females as males, some have found the opposite,^35^, ^36^ whilst the majority of studies have found no differences between males and females with DDD^3^, ^5^, ^21^, ^27^, ^37^, ^38^, ^39^, ^40^, ^41^ or C3GN.^21^, ^42^ Native renal manifestations of C3 glomerulopathy are diverse and nonspecific, with hypertension, hematuria, proteinuria (with or without nephrotic syndrome), and renal failure all reported. Most studies have found no difference in presenting features between DDD and MPGN type 1.^28^, ^29^, ^32^, ^38^, ^40^ However, nephrotic syndrome was approximately twice as common in MPGN type 1 as either DDD or C3GN in the recently updated French C3 glomerulopathy series.^21^

In CFHR5 nephropathy, persistent microscopic hematuria with or without episodes of synpharyngitic macroscopic hematuria (clinically identical to those seen in IgA nephropathy^43^) is the usual presentation. Identical features occasionally have been reported in DDD patients,^34^, ^44^ in whom the clinical and histologic overlap with acute postinfectious GN suggests that intercurrent infection is important in renal injury when AP dysregulation is present. Thus, symptoms of respiratory tract infection preceding renal symptoms have been described in children (not usually adults) with DDD,^4^, ^5^, ^17^, ^35^, ^38^, ^39^, ^40^, ^41^, ^45^, ^46^ sometimes in association with increased anti–streptolysin O titers.^17^, ^38^, ^41^, ^46^

Nonrenal manifestations of DDD include ocular drusen, acquired partial lipodystrophy (PLD), type 1 diabetes mellitus, and monoclonal gammopathy of undetermined significance (MGUS). Ocular drusen are lipoproteinaceous deposits of complement-containing debris between the basal surface of the retinal pigment endothelium and Bruch’s membrane. The clinical association of ocular drusen with DDD was first reported in 1989,^47^ and appears to be related to structural and functional similarities between Bruch’s membrane, a multilaminar extracellular matrix that separates the retina from the blood vessels in the choroid, and the GBM. Ocular drusen are also a manifestation of age-related macular degeneration (AMD),^48^ the leading cause of blindness in the industrialized world, for which a number of shared genetic risk factors with DDD are discussed later. However, unlike AMD, drusen in patients with DDD are said to occur at a young age and to be associated with only a modest risk of progressive visual loss.^49^ In the recent therapeutic trial of eculizumab in DDD and C3GN, among several patients identified as having ocular drusen, one patient had C3GN.^50^

PLD involves loss of subcutaneous fat from the face and upper body. An association with GN was first noted in 1958.^51^ Early reports of PLD in patients with DDD^52^ and C3NeF-associated MPGN (without EM)^53^ were followed by several MPGN cohorts in which PLD was associated specifically with DDD.^29^, ^31^, ^38^ In the updated French C3 glomerulopathy series, PLD was noted only in the DDD group,^21^ affecting 17% of DDD patients (versus <5% in other recent DDD cohorts^26^, ^54^). However, in an early PLD series comprising six patients with MPGN (all having C3NeF and low serum C3 levels), one patient had only subendothelial deposits on EM.^55^ Moreover, the Necker hospital group reported two patients with PLD and MPGN in whom IF was positive for both C3 and IgG.^56^ In a 2004 literature review of PLD, MPGN was the most common associated renal lesion, but only slightly more than half of MPGN cases were shown to be DDD, with MPGN types 1/3 also represented.^57^ In the majority of cases of PLD, including those associated with low serum C3 levels, renal disease was not found.^57^ A significantly increased risk of diabetes mellitus type 1 was reported in patients and families with DDD in a US questionnaire-based study of 98 patients (although ascertainment bias cannot be excluded).^39^ It has been suggested that MGUS is associated with DDD^4^, ^58^, ^59^ and C3GN,^18^, ^60^ and that it may confer a poor renal prognosis.

## Prognosis

Progression to end-stage kidney disease (ESKD), despite treatment, is said to occur in approximately half of patients with a diagnosis of DDD of 10 or more years.^31^, ^34^, ^49^, ^61^, ^62^ In the recently updated French C3 glomerulopathy series, overall 10-year progression to ESKD was 36.5% (with a mean follow-up period of 11 years).^21^ No significant difference was seen between the three diagnostic categories (DDD, C3GN, and MPGN type 1). This is consistent with the finding in earlier MPGN cohort studies that DDD was not more likely than other forms of MPGN to progress to ESKD.^7^, ^29^, ^31^, ^40^, ^63^, ^64^, ^65^ Other series found that an apparent increase in ESKD rates probably was confounded by a higher incidence of crescents^30^, ^38^ or reduced GFR^61^ at diagnosis in the DDD versus non-DDD patient groups.

The impact of clinical or histologic variables on outcomes of C3 glomerulopathy has been assessed in numerous retrospective cohort studies. In the two most recent DDD cohorts, reduced GFR at diagnosis was associated significantly on multivariate analysis with progression to ESKD.^4^, ^21^ In CFHR5 nephropathy, in which progressive disease is characterized by hypertension, proteinuria, and chronic renal failure, ESKD has been reported in 18 of 91 carriers (or 20%) of the heterozygous *CFHR5* gene mutation. Of these, 14 were men and 4 were women, a difference that remains unexplained. No other features have been shown consistently to correlate with disease outcomes in DDD or other forms of C3 glomerulopathy. This is partly because of the limited number of end points that can be assessed in mostly small cohorts. In the updated French C3 glomerulopathy cohort,^21^ adults with DDD had significantly reduced renal survival compared with children with DDD, adults with C3GN, and adults with MPGN type 1. However, the DDD adult subgroup comprised only 11 patients, and because other differences between these subgroups were not assessed it is difficult to determine if this was a robust conclusion. For some variables, including the presence of crescents on the diagnostic biopsy specimen (found to predict a rapid progression to ESKD in some DDD cohorts^38^, ^66^ but not others^4^, ^29^, ^35^), it now seems especially clear that prognostic value cannot be assessed adequately through retrospective cohort analyses. This is exemplified by the US DDD cohort study in which it was suggested that the reason crescents did not correlate with outcomes may have been the increased use of immunosuppressants in such patients.^4^

## Pathogenesis

### C3 Nephritic Factors

Reduced serum C3 (but not C4) is consistent with AP dysregulation, but is neither sensitive nor specific for the diagnosis of DDD/C3GN. In recent cohort studies, isolated low C3 level was found in only a proportion of patients with DDD^4^, ^21^ and C3GN.^21^, ^42^ C3NeF, first described in a patient with “persistent hypocomplementemic glomerulonephritis,”^67^ is an autoantibody that binds to a neoepitope on C3bBb, the C3 convertase of the AP, stabilizing it against complement factor H (Cfh)-mediated decay and prolonging its C3-cleaving action.^68^ A second type of C3NeF was found to display slower C3 and C5 activation^69^ and dependence on properdin for convertase stabilization.^70^ It has been reported in very small series that properdin-dependent C3NeFs are more characteristic of MPGN types 1^70^, ^71^ and 3,^70^ whereas properdin-independent C3NeFs are found more often in DDD and PLD.^71^

In the updated French C3 glomerulopathy cohort,^21^ C3NeFs were significantly more common in patients with DDD than C3GN or MPGN type 1 (consistent with earlier studies^29^, ^31^, ^72^). Patients with C3NeFs also had generally lower C3 levels (although C3 was normal in ~40% of patients with C3NeF). Here, as in most DDD/C3GN studies, serum C3 levels and the presence of C3NeF did not correlate with disease outcomes. The identification of C3NeFs in patients with other forms of GN and in healthy individuals (discussed in one recent study^73^) underlines a lack of specificity. In CFHR5 nephropathy, C3 levels typically are normal and C3NeF is absent.^74^

Autoantibodies that bind to native factor B (Cfb) and stabilize the AP C3 convertase^75^ or target both Cfb and C3b^76^ also have been described in patients with DDD. An early report showed that circulating monoclonal Ig λ light chain dimers were able to activate the AP.^77^ The λ light chain dimers were found to bind short consensus repeat (SCR) 3 on the Cfh molecule, blocking one of its C3b interaction sites.^78^ Subsequently, anti-Cfh autoantibodies have been identified in patients with DDD^59^, ^73^, ^79^ and C3GN.^17^, ^18^

### Family Studies

**Figure 3:**
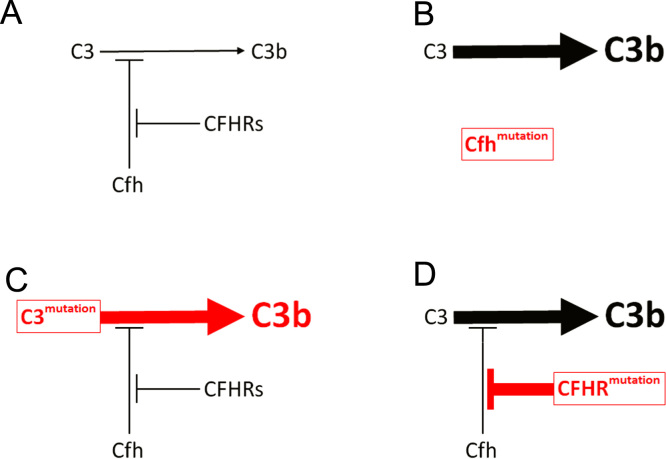
Disease mechanisms in C3 glomerulopathy, based on genetic defects identified in family studies. (A) Physiological regulation of C3 activation to C3b via the alternative pathway is mediated by Cfh. Competitive inhibition of Cfh by CFHR proteins is termed *Cfh deregulation*. (B) Homozygous deficiency or dysfunction of Cfh results in excessive C3 activation. (C) Hyperfunctional C3 produces excessive C3 activation despite normal Cfh activity. (D) Abnormal CFHR proteins enhance Cfh deregulation, leading to excessive C3 activation.

Renal biopsy data for a number of family studies in C3 glomerulopathy are shown in Table 1. These studies indicate a genetic basis for a small number of C3 glomerulopathy cases, although the genetic defect has been characterized and an underlying pathogenic mechanism proposed in only a handful of families. These include cases associated with homozygous Cfh deficiency or homozygous loss-of-function *Cfh* mutation, heterozygous gain-of-function *C3* mutation, and *CFHR* mutations leading to enhanced Cfh deregulation (summarized later and in Figure 3). Two Algerian brothers of consanguineous parents were reported with severe Cfh deficiency and autosomal-recessive “atypical DDD” as shown on EM by discontinuous intramembranous deposits.^44^ Renal disease presented during infancy with intermittent macroscopic hematuria and infections (including pharyngitis) together with failure to thrive and developmental delay in the younger sibling. Both brothers were negative for C3NeF but had very low serum levels of C3, Cfb, properdin, and C5 with normal classic pathway components. A homozygous missense mutation in SCR 7 of the *Cfh* gene, causing a substitution of a cysteine with a serine residue, subsequently was identified in the elder brother.^80^ Both parents and two other children without renal disease had half-normal Cfh levels, consistent with heterozygosity. A young girl presenting with recurrent macroscopic hematuria and undetectable serum C3, Cfb, and Cfh was found to have C3GN with a mesangioproliferative pattern and predominant mesangial deposits on EM.^81^ A homozygous missense mutation was detected on SCR 16 of *Cfh* that resulted in a cysteine to serine change. Both unaffected parents were heterozygous for the *Cfh* mutation and displayed low-normal Cfh levels, while an unaffected sister had two normal *Cfh* alleles and high-normal Cfh levels. Intriguingly, an earlier report concerned a patient with severe Cfh deficiency in association with type 3 collagen glomerulopathy^82^ (more likely representing chronic thrombotic microangiopathy^16^) in whom a different homozygous missense mutation was identified at the same site on SCR 16.^83^ The substitution of a cysteine residue (in this case with a tyrosine) altered the secondary structure of the Cfh protein, resulting in its nonsecretion.^84^ A young girl was reported with macroscopic hematuria, very low C3 and Cfh levels, and endocapillary GN with predominant glomerular C3.^85^ A homozygous missense mutation on SCR 2, causing an exchange of proline to serine, was apparently the result of paternal isodisomy. The patient also was homozygous for a risk haplotype for the membrane cofactor protein (MCP) gene (discussed later).^86^

**Table 1:** Renal Biopsy Data in Family Studies of C3 Glomerulopathy

Two young daughters of consanguineous Turkish parents had C3GN with low C3, positive C3NeF, and only slightly reduced serum Cfh levels.^87^, ^88^ A loss-of-function homozygous mutation in *Cfh* involving deletion of a single codon in SCR 4 was found to produce a circulating mutant Cfh protein with impaired binding to C3b, defective decay accelerating activity, and reduced factor I (Cfi) cofactor activity. Both patients also were homozygous for the DDD/AMD-associated Y402H *Cfh* polymorphism (discussed later). A mother and her twin adult sons had DDD in association with half-normal C3 and Cfb levels and normal Cfh levels.^89^ A gain-of-function heterozygous mutation of the *C3* gene involving deletion of two codons was found to produce a hyperfunctional C3 protein. Mutant C3 was unable to be activated by the C3 convertase of the AP. However, after its conversion by proteases to C3b or by spontaneous hydrolysis of the internal thioester (C3 tickover) to C3(H_2_O), it formed a C3 convertase that was resistant to regulation by Cfh. This resulted in accelerated breakdown of wild-type C3 that appeared to be restricted to the fluid phase, because regulation in vitro by decay accelerating factor (DAF, a surface-expressed complement regulatory protein) was intact. Similarly, mutant C3b and C3(H_2_O) did not undergo Cfi-mediated proteolysis in the fluid phase (being dependent on Cfh cofactor activity), whereas proteolysis proceeded efficiently in the presence of MCP.

In CFHR5 nephropathy, multiplex-ligation probe amplification enabled the identification of a novel heterozygous mutation in the *CFHR5* gene involving internal duplication of the initial two N-terminal SCRs in two affected families originally from the Troodos region of Cyprus.^74^ The finding of a common mutation in these families suggested a founder effect, and subsequently diagnostic polymerase chain reaction enabled the identification of numerous additional affected individuals and families from all over Cyprus (some with a previous diagnosis of MPGN type 1). A total of 91 carriers of the same heterozygous founder mutation from 16 apparently unrelated Greek-Cypriot families was later reported, with a disease penetrance of 90%.^90^ An Irish family was first reported with autosomal-dominant MPGN type 3^91^ (now reclassified as C3 glomerulopathy) and linkage to the regulators of complement activation locus.^92^ A novel hybrid *CFHR3-1* gene now has been identified encoding an abnormal CFHR3-1 protein in affected family members.^93^

### Cfh Deregulation

Until recently, the biological role of the five CFHR proteins has been unclear, although the presence of polymorphisms involving homozygous deletion of *CFHR* genes (most commonly *ΔCFHR3/1*)^102^ in healthy populations suggested that the CFHR3 and CFHR1 proteins were nonessential. Recently, CFHR1, CFHR2, and CFHR5 have been shown to possess a common dimerization motif located within the highly conserved first two N-terminal SCRs of these particular CFHR proteins. It is now believed that, unlike Cfh, these CFHR proteins do not have any complement inhibiting actions in vivo. Rather, they compete with Cfh for binding to C3b, thereby enhancing C3 activation in a process termed *Cfh deregulation*.^103^ Both dimerization and deregulation have implications for understanding how CFHR proteins mediate autosomal-dominant C3 glomerulopathy. In CFHR5 nephropathy, the mutation results in the duplication of the dimerization motif and enables generation of large complexes that have enhanced deregulation.^103^ Enhanced deregulation also has been shown using serum containing the abnormal (hybrid) CFHR3-1 protein.^103^ The current paradigm is that there is either continuous or intermittent (eg, during infection) activation of C3 along the GBM that is controlled by Cfh. In the presence of abnormal CFHR proteins, enhanced deregulation of Cfh enables C3 to accumulate along the GBM. This paradigm would predict that a lack of CFHR proteins (eg, through the common *ΔCFHR3/1* allele) would enhance Cfh action and become a protective factor in C3 glomerulopathy or complement-mediated disease in general. It also may explain the protective effect of *ΔCFHR3/1* in AMD^102^, ^104^ and IgA nephropathy.^105^ Homozygous *ΔCFHR3/1* was absent in one cohort of 68 DDD patients, despite a rate among healthy control subjects of 3%.^26^

### Other Genetic Associations

Additional genetic variations in complement proteins and/or regulators have been identified using a candidate gene approach in individuals and cohorts with C3 glomerulopathy. Most of those in whom a rare genetic variant has been found have a family history of disease (and hence a higher pretest probability of genetically determined susceptibility). The relatively poor yield of genetic screening to date in nonfamilial cases is exemplified by the French C3 glomerulopathy cohort, in which mutations in one of three genes (*Cfh, Cfi*, and *MCP*) was reported in only 18% of all patients screened.^21^ *Cfh* mutations were the most common, occurring in 9 of 16 individuals with a family history, but in only 8 nonfamilial cases. Another remarkable finding in this study was of no overall difference in the proportion of patients with DDD, C3GN, and MPGN type 1 in whom mutations affecting complement regulatory proteins were found. Thus, patients with homozygous *Cfh* mutations were identified in DDD (as outlined earlier), C3GN (patient 3^106^), and MPGN type 1 (patient 2^106^ and case 1^107^). However, the bulk of mutations identified in the French cohort were heterozygous. Two patients treated with eculizumab in the recent therapeutic trial also had heterozygous mutations affecting *Cfh* and *MCP*.^50^ Unlike homozygous Cfh deficiency, for which an animal model exists (discussed later), the functional basis of heterozygous mutations in C3 glomerulopathy remains unclear, notwithstanding that some have shown a correlation with AP dysregulation in patients with aHUS. Of note, homozygous Cfi deficiency, first reported in a patient with extremely low C3 and increased susceptibility to infection,^108^ has not been reported in association with C3 glomerulopathy.

Screening of patient cohorts, followed by comparison with control populations, also identified common allelic variants that may modify risk of C3 glomerulopathy. Multiple genetic variants potentially conferring either increased risk or protection for C3 glomerulopathy and/or other complement-related phenotypes are said to define an individual’s complotype. Thus, single-nucleotide polymorphisms (SNPs) in the *Cfh* and *C3* genes and a deletional copy number variation in *C4A* are associated with increased risk of DDD, whereas an SNP in *CFHR5* is protective.^109^ An SNP in *MCP* also is associated with an increased risk of C3GN and MPGN type 1.^21^ At-risk haplotypes combining multiple SNPs in *Cfh*^110^ and *C3*^109^ have been delineated for DDD, together with a protective *Cfh* haplotype.^110^ An at-risk *MCP* haplotype for C3GN and MPGN type 1 and a protective *MCP* haplotype for DDD and C3GN also have been reported.^21^ In some, but not all, cases, functional studies showing AP dysregulation have provided evidence in support of the genetic association.^111^

In AMD, approximately 50% of the population-attributable risk is accounted for by common polymorphisms in the *Cfh* gene. Several AMD-associated *Cfh* sequence variants have been shown to confer a common susceptibility to DDD.^112^ One important example is the Y402H substitution of a histidine (H) for a tyrosine (Y) residue on SCR 7, strongly associated with both AMD^110^, ^112^ and DDD^110^ in Caucasian but not Asian populations. Functional studies have indicated that Y402H affects differential binding of Cfh to glycosaminoglycans within Bruch’s membrane and the glomerulus.^113^ An illustrative case relates to a 56-year-old man in whom ESKD developed as a result of DDD (without diagnostic EM), followed by new-onset AMD, causing eventual blindness.^114^ In this individual, genetic susceptibility to AMD and DDD derived from a heterozygous Cfh mutation in SCR 7, a homozygous risk haplotype comprising the H402 variant for SCR 7, and several other AMD risk factors.

### Animal Models

Pigs^115^ and mice^116^, ^117^, ^118^, ^119^, ^120^ with homozygous Cfh deficiency have provided an important experimental model of DDD. In Norwegian Yorkshire piglets (now extinct), a lethal *Cfh* mutation occurred spontaneously and was associated with severe renal disease described as porcine MPGN type 2. Gene-targeted Cfh-deficient mice show AP dysregulation with extremely low plasma C3 levels.^116^ *Cfh−/−* mice develop albuminuria and MPGN, with significantly reduced survival compared with wild-type mice. In both models glomerular C3 without Ig is seen at an early stage and precedes the appearance on EM of capillary wall dense deposits. These electron-dense deposits are initially subendothelial rather than intramembranous, and show positive staining for C3 using immunoelectron microscopy. The absence of extraglomerular C3 is reminiscent of “atypical DDD,” as described in the family study of homozygous Cfh deficiency.^44^ *Cfh−/−* mice crossed with Cfb-deficient mice do not show low plasma C3 levels or glomerular changes, indicating that uncontrolled C3 activation via the AP is critical to renal pathogenesis. Injection into *Cfh−/−* mice of a single dose of purified murine^119^ or human^120^ Cfh restores plasma C3 levels to normal at 24 hours, accompanied by the resolution on IF of GBM C3 staining and the appearance of mesangial C3. In affected piglets, weekly plasma infusions containing Cfh also reduced the severity of GN and improved survival.^121^

The role of C5 in this phenotype was studied by crossing *Cfh−/−* mice with those lacking C5.^117^ Although spontaneous MPGN with capillary wall deposits was still observed, glomerular cellularity/crescents were reduced with improved renal function and reduced mortality. *Cfh−/−* mice with accompanying C5 deficiency also were less susceptible to renal injury induced by sheep nephrotoxic serum, whereas *Cfh−/−* mice with or without accompanying C6 deficiency showed an influx of neutrophils into the glomerulus. This suggests that the production of C5a rather than membrane attack complex (MAC) formation mediated renal damage. *Cfh−/−* mice injected with anti-C5 antibodies before administration of nephrotoxic serum also were protected, suggesting that anti-C5 therapy may be beneficial in human beings with C3 glomerulopathy owing to Cfh deficiency (eg, during disease flares). In mice with homozygous deficiency of Cfi, an inability to metabolize C3b results in uncontrolled AP activation and low circulating C3 levels. In *Cfi−/−* mice only C3b is evident in plasma, consistent with the C3 profile in human beings with total Cfi deficiency.^122^ *Cfi−/−* mice show mesangial C3 accumulation and nodular mesangial expansion, but without GBM C3 staining or features of MPGN. Unexpectedly, in mice with combined homozygous deficiency of Cfh and Cfi, both the plasma C3 profile and glomerular C3 pattern are identical to those seen in Cfi deficiency alone.^118^ Injection into *Cfh−/−.Cfi−/−* mice of a source of murine Cfi resulted in cleavage of C3b, generating fragments including iC3b. This was accompanied by loss of mesangial C3 staining, and the appearance of GBM C3 staining. It therefore appears that cleavage of C3b was critical for GBM C3 staining to occur, and that measures to prevent conversion of C3b to iC3b might be useful therapeutically in patients in whom GBM C3 accumulation is shown.

Recently, an additional aspect of renal pathogenesis was elucidated through the generation of mice with Cfh and properdin deficiency.^123^, ^124^ Because properdin prolongs the cleaving activity of the AP C3 convertase, it was hypothesized that genetic deficiency of properdin (as a positive regulator of the AP) would ameliorate the renal phenotype of Cfh deficiency. Unexpectedly, *Cfh−/−.P−/−* mice showed more severe MPGN and more intense GBM C3 staining than *Cfh−/−* mice.^123^ Plasma C3 levels did not differ between *Cfh−/−.P−/−* and *Cfh−/−* mice, although only *Cfh−/−.P−/−* mice showed (fractional amounts of) intact C3. Plasma C5 was absent in *Cfh−/−* mice (consistent with previous data^125^), but was detected at low levels in *Cfh−/−.P−/−* mice. One explanation for these observations is that higher levels of intact C3 in *Cfh−/−.P−/−* mice may increase the availability of iC3b in the circulation for accumulation along the GBM, whereas the increased availability of C5 also may contribute to the more severe glomerular inflammation seen in these mice. Although the predominant C3 depletion seen in *Cfh−/−.P−/−* mice closely resembles the C3 profile in human subjects with properdin-independent C3NeFs, the pattern of C3 and C5 depletion in *Cfh−/−* mice is analogous to properdin-dependent C3NeFs.

## Treatment

No treatment has been proven to be beneficial in C3 glomerulopathy.

Antihypertensive/antiproteinuric therapy, particularly via renin-angiotensin-aldosterone system (RAAS) blockade with angiotensin-converting enzyme inhibitors or angiotensin-receptor blockers, generally is recommended in GN.^126^ Evidence in support of RAAS blockade in C3 glomerulopathy specifically is limited to retrospective cohort studies (which are subject to treatment bias). In the French C3 glomerulopathy cohort, RAAS blockade, but not use of immunosuppressants, was associated with a significant increase in renal survival,^21^ whereas in the US DDD cohort a nonsignificant reduction in progression to ESKD was found with combined RAAS blockade and steroids.^4^ After early reports of children with DDD in whom alternate-day steroids were associated with histologic regression,^127^ a 1992 randomized controlled trial showed clinical benefit in 70 children with MPGN, of whom 14 had DDD (without a subgroup analysis).^128^ In the absence of subsequent randomized controlled trials, reports purportedly showing response to steroids in DDD are complicated by the highly variable natural history, which includes rare occurrences of sustained clinical remission even without treatment.^129^, ^130^ Thus, in one pediatric series of likely C3 glomerulopathy, treatment with steroids, initiated on the basis of biopsy evidence of acute glomerular inflammation including crescents, led to clinical improvement in a number of cases.^131^ However, an identical improvement was seen in an untreated patient. Use of steroids and immunosuppressants may appear most justified in patients with C3 glomerulopathy who develop rapidly progressive GN (because this would prompt their use for other causes of GN). However, post-transplant recurrence of C3 glomerulopathy (including with crescents on biopsy, discussed later) and aHUS^132^ suggests that immunosuppression (as used in the transplant period) may be ineffective for AP-mediated renal disease. The iatrogenic risk of infection, potentially triggering complement activation and exacerbation of nephritis, also must be weighed. Treatment with the anti-CD20 monoclonal antibody rituximab has been reported with success in a single case of DDD related to anti-Cfb/C3b autoantibodies.^76^ Immunosuppressive treatment for underlying hematologic malignancies including MGUS, in patients with associated C3 glomerulopathy, leading to improvement in renal parameters in some reports^60^ but not others,^18^ is not discussed here.

Restoration of normal AP regulation is a major goal of current research into treatment of C3 glomerulopathy. The animal models suggest that purified or recombinant Cfh replacement might be beneficial in Cfh-deficient patients. Although this therapy is not currently available, long-term plasma infusion was effective and well tolerated in the sisters with familial C3GN related to circulating mutant Cfh.^87^ Elsewhere plasma infusion/exchange has been reported with only mixed success, including in the setting of recurrent post-transplant DDD.^133^ A therapeutic role for anti-C5/C5a therapy in C3 glomerulopathy flares is suggested by experimental data in animal models and by the observation in DDD patients with rapidly progressive GN that the glomerular deposits contain C5.^23^ The C5-inhibitor eculizumab has been reported with success in several cases of DDD^134^, ^135^ (including a patient with post-transplant recurrence associated with renal failure^136^) and one of MPGN type 1.^137^ However, it also has been reported with failure in another DDD patient,^134^ and with mixed results in a recent prospective, uncontrolled trial in 6 adults with native or recurrent post-transplant DDD or C3GN.^50^ At the conclusion of a 1-year course of eculizumab, a clinical improvement was observed in three of the trial patients (two showing reduced inflammation on biopsy^138^). In the future, agents that either inhibit the C3 convertase of the AP or remove C3 (and/or C3 fragments) from the circulation may undergo evaluation (bearing in mind the key protective functions these molecules also perform in innate immunity).

## Transplantation

Histologic recurrence of DDD after renal transplantation was first reported by Galle et al^139^ and is common.^140^ Histologic recurrence of C3GN probably also was described in older studies,^141^ with recent reports^21^, ^42^ including cases of CFHR5 nephropathy^142^ and the *CFHR3/1* hybrid gene (in which the related donor also later developed C3 glomerulopathy).^91^ A US retrospective cohort of almost 8,000 pediatric transplant recipients, including 75 children with DDD, assessed the impact of histologic recurrence of DDD on graft survival.^6^ At 5 years, 50% of pediatric DDD recipients had viable grafts (comparable with the 45% rate identified in an accompanying literature review of 78 pediatric and adult DDD recipients). This was significantly lower than the pediatric graft survival rate of 74% in all-cause ESKD and 72% in non-DDD forms of GN. Of 29 graft failures in DDD recipients during the study period, 11 were attributed to recurrent disease (9 after deceased donor transplantation). However, the contribution of recurrent disease to the overall increase in pediatric graft failure rates in DDD recipients was not assessed, except in a subgroup of 29 of the DDD recipients. Here, 4 graft losses resulting from biopsy-proven recurrent disease represented a nonsignificant reduction in median graft survival. Of note, crescents were seen on graft biopsy only in those with evidence of DDD recurrence, and were significantly associated with reduced graft survival. Biopsy-proven recurrence was always accompanied by heavy proteinuria, but no clinical or laboratory variables predicted either DDD recurrence or graft loss. The investigators concluded that there was no correlation between the isolated recurrence of dense deposits and reduced graft survival.^6^

More recent studies include an Irish cohort of 33 patients with primary idiopathic MPGN in whom graft failure rates were reported only for those with biopsy-proven recurrence.^30^ This included 11 of a total of 18 transplants performed in recipients with (nonfamilial) DDD. The investigators concluded that 14 grafts lost to recurrent MPGN at a median of 7.5 years was likely to represent a reduction in graft half-life.^30^ In the US questionnaire-based DDD study comprising 22 mostly pediatric transplant recipients, 10 grafts were lost within the first 5 years (7 were attributed to recurrent disease).^37^ In one C3GN cohort, post-transplant recurrence in two individuals was heralded by increased creatinine levels, subnephrotic proteinuria, and low serum C3 levels.^42^ In CFHR nephropathy, 10 patients (mostly males) have been reported with successful transplantation (including a recipient whose female related donor was an asymptomatic carrier of the mutation), despite histologic recurrence in all three patients who underwent a biopsy.^90^, ^142^

## Conclusions

DDD is distinguished histologically from other forms of C3 glomerulopathy by the presence of osmiophilic, intramembranous, ribbon-like deposits. Certain clinical features, including a young age at diagnosis and (in rare cases) an association with ocular drusen, are more typical of DDD than other forms of C3 glomerulopathy. A diagnosis of DDD does not appear to confer a worse renal prognosis compared with other forms of C3 glomerulopathy. Evidence for the role of AP dysregulation in C3 glomerulopathy is drawn from linkage studies in a small number of pedigrees, cohort studies showing an association with C3NeFs and complement regulatory gene variants, and animal models. Recently, linkage was shown for copy number variation across different *CFHR* genes in familial C3 glomerulopathy, and a new pathophysiologic concept of Cfh deregulation has been proposed. Because progression to ESKD and post-transplant recurrence is common in all forms of C3 glomerulopathy, therapies that target underlying disease mechanisms are urgently required.^91^, ^92^, ^93^, ^94^, ^95^, ^96^, ^97^, ^98^, ^99^, ^100^, ^101^
